# Value of Negative Eugenics

**Published:** 1916-01

**Authors:** Edwin G. Conklin

**Affiliations:** Professor of Biology, Princeton University, Princeton, N. J.


### Value of Negative Eugenics.

BY EDWIN G. CONKLIN, PROFESSOR OF BIOLOGY, PRINCETON UNIVERSITY, PRINCETON, N. J.

No well-informed person doubts that the principles of heredity and evolution apply to man as well as to the lower organisms, and in spite of much controversy with respect to the importance of natural selection in evolution I make bold to assert that no other principle has yet been suggested of equal importance with this, and that the elimination of the unfit affords not only the only natural explanation for the existence of fitness, but also the only means by which breeders have been able to improve domesticated animals and cultivated plants. The only possible control which mankind can exercise over the production of improved races of lower organisms or of men lies in the elimination from reproduction of the less favorable variations which are furnished by nature. For it has become more and more clear in recent years that, while environment exercises a great influence over the development of the individual, its influence on the germ plasm or the hereditary characteristic of the race is relatively slight and in general is not of a definite or a specific character. Probably environment may under certain circumstances modify the germ plasm, but there is no evidence that good environment will produce good modifications, and bad environment bad modifications in this hereditary substance. Consequently, the only method which is left to man for improving races is found in sorting out the favorable varieties from the unfavorable ones which are furnished by nature. If the human race is to be permanently improved in its inherited characteristics, there is no doubt that it must be accomplished in the same way in which man has made improvements in the various races of domesticated animals and cultivated plants.

Fortunately, or unfortunately, the methods which breeders use cannot be rigidly applied in the case of man. It is possible for breeders to eliminate from reproduction all except the very best stocks, and this is really essential if evolution is to be guided in a definite direction. If only the very worst are eliminated in each generation, the standard of a race is merely maintained, but the more severe the elimination is, the more does it become a directing factor in evolution. This may be illustrated by a diagram in which variations in all directions are represented by lines radiating from a central point. These lines may be thought of as being individually distinct, as in the "pure line" concept. If only those lines are blocked which lead in one direction, the center of radiation or "mode" would be but slightly changed in successive generations. But if. all lines are blocked but those which lead in one particular direction, the mode will be rapidly shifted in that direction in succeeding generations. Therefore the value of selection as a directing factor in evolution depends on its severity.

### MAINTAINING THE LEVEL.

In the case of man, however, even the most enthusiastic eugenicists have never proposed to cut off from the possibility of reproduction all human stocks except the very best, and if only the very worst stocks are thus eliminated, we must face the conclusion that practically all that can be accomplished will be to preserve the race at its present level. The ecstatic visions of those eugenicists who look forward to a world of supermen to be produced by this method of eliminating from reproduction the worst human stocks, can be regarded only as iridescent dreams, impossible of fulfillment. It is impossible, then, to apply rigidly to man the methods of animal and plant breeders. Society cannot be expected to eliminate from reproduction all but the very best lines. The great majority of mankind cannot be expected voluntarily to efface itself. The most that can be hoped for is that the great mediocre majority may eliminate from reproduction a very small minority of the worst individuals.

Furthermore, other and perhaps even more serious objections to the views of extreme eugenicists are to be found in human ideals of morality. Even for the laudable purpose of producing a race of supermen, mankind will probably never consent to be reduced to the morality of the breeding-pen with a total disregard of marriage and monogamy. The geneticist who has dealt only with chickens or rabbits or cattle is apt to overlook the vast difference between controlling reproduction in lower animals and in the case of man where restraints must be self-imposed.

Another fundamental difficulty in breeding a better race of men is to be found in a lack of uniform ideals. A breeder of domestic animals lives long enough to develop certain races and see them well established, but the devotee of eugenics cannot be sure that his or her ideals will be followed in succeeding generations. The father of Simon Newcomb is said to have walked through the length and breadth of Nova Scotia seeking for himself a suitable mate, but neither he nor any other eugenicist could be sure that his descendants would follow a similar course, and long continued selection along particular lines must be practiced if the race is to be permanently improved. Mankind is such a mongrel mixture, and it is so impracticable to exercise a strict control over the breeding of men, that it is hopeless to expect to get pure homozygous stocks except with respect to a very few characters and then only after long selection.

But granting all these difficulties which confront the eugenicist, there is no doubt that something may be gained by eliminating the worst human kinds from the possibility of reproduction, even though no great improvement in the human race can be expected as a result of such a feeble measure. The question which has been assigned to me on this occasion is "How the Number of Births of Children Receiving a Faulty Heritage from their Parents May Be Reduced?" Strictly speaking, there is no one who does not receive a faulty heritage, at least in some respects; "there is none perfect, no not one." But there are some whose heritage is so faulty that they constitute a menace to society, and it is doubtless to these that this question refers. There are large numbers of persons, loosely called "defectives," in modern society, and it seems to be a question whether they are not actually increasing in number. This increase may be due, however, to a more accurate recognition and classification of defectives than prevailed formerly. There is no clearly and sharply defined class of defectives, but human populations show every gradation from the highest and most efficient individuals to the lowest and worst; strictly speaking, defectives may be said to include all individuals below the average, from subnormals to monsters. In general all defectives are shorter lived than normals, and the more serious the defect the shorter the life. The worst monstrosities die in the early stages of development, others live but a short time after birth, and none of these ever leave offspring. Only those defectives in whom abnormalities are relatively slight ever reproduce. Nature has thus erected an insuperable barrier against the propagation of the worst.

ONE EFFECT OF CHARITY.

Nevertheless a good many defectives survive in modern society and are capable of reproduction who would have perished in more primitive society before reaching maturity. In the most highly civilized States the lives of these unfortunates are preserved by charity, and in not a few they are allowed to reproduce, and thus natural selection, the great law of evolution and progress, is set at naught. It is within the power of society to eliminate from reproduction this dependent class.

How can the number of defectives born from defective parents be reduced? Evidently if these defects are hereditary it can be done only by preventing their breeding, since in modern society defectives cannot be destroyed by Spartan methods. Many ways have been recommended and a few have been tried to accomplish this end, but they all come under two categories: (1) Segregation to prevent the union of the sexes; (2) sterilization or other means to prevent conception following sexual union. Such methods if rigidly applied to all defective or abnormal persons would doubtless reduce the number of "children receiving a faulty heritage from their parents," but since it is impossible for reasons indicated above to apply these methods to any except the most seriously defective class, which is usually dependent upon public care or private charity, and since in general the birth rate at present among such defectives is not large, no great change in the number of births of defective children through such elimination need be anticipated. And this is especially true since children inherit not merely the traits which their parents show, but also those family traits which are carried along in the germ plasm in a latent or recessive form, waiting only for an opportunity to become patent. The study of heredity shows that the normal brothers and sisters, or even more distant relatives, of defective persons may carry the defect in their germ plasm and may transmit it to their descendants though not showing it themselves. Such persons are more dangerous to society than the defectives themselves. And yet it is probably impossible rigidly to exclude them from reproduction.

Finally, it is usually difficult and often impossible to decide whether a given defect is due to heredity or to environment; if it is due to the latter the methods adopted for its prevention must be wholly different from what they would be if it is due to the former cause. Experiments on guinea pigs, rabbits, and other animals show that serious defects may be produced in offspring by the action of alcohol and drugs on either or both of the parents before conception, and Forel with his wide experience in such matters does not hesitate to maintain that the effect of alcohol on either or both of the parents at the time of conception is one of the most fruitful causes of monstrous or defective children. No doubt there are many other environmental causes of defects in children, such as infection, malnutrition, injury, etc., at various stages in their development.

Dr. Henry H. Goddard, of the Training School at Vineland, N. J., has kindly furnished me with the following figures regarding the mental condition, so far as it has been investigated, of the parents of the inmates of that institution:

| | | |
|---|---|---|
| Number of families investigated | 337 or | 100% |
| Both parents feeble-minded | 57 or | 16.9% |
| One parent feeble-minded and other normal | 43 or | 12.8% |
| One parent feeble-minded and other unknown | 54 or | 16.0% |
| Family history of feeble-mindedness | 154 or | 45.7% |
| Both parents normal | 90 or | 26.7% |
| One parent normal, the other unknown | 47 or | 13.95% |
| Both parents unknown | 46 or | 13.65% |

I am indebted to Dr. Martin W. Barr of the Pennsylvania Training School at Elwyn, Pa., for an extensive etiological table which he has prepared showing the probable causes of mental defect in more than 4000 cases, from which I quote the following summaries:

| | No. of cases. | Percentage |
|---|---|---|
| Causes acting before birth | 2,651 | 65.45 |
| Family history of idiocy and imbecility ... 1,030 or 25.43% | | |
| Causes acting at birth | 186 | 4.59 |
| Causes acting after birth | 1,213 | 29.96 |
| Totals | 4,050 | 100. |

Dr. George Mogridge, Superintendent of the Iowa Institution for Feeble-Minded Children, has kindly supplied me with the following figures regarding the reported mental condition of the parents of persons who have been admitted to that institution, at the same time warning me that such statistics are not altogether reliable:

| | | | |
|---|---|---|---|
| Number of families investigated | 1,701 | or | 100.0% |
| Inmates who have both parents feeble-minded | 66 | or | 3.88% |
| One parent feeble-minded and other normal or unknown | 134 | or | 7.88% |
| Number with both parents normal | 513 | or | 30.16% |
| Number with both parents unknown | 876 | or | 51.5% |
| Insanity in one or both parents | 112 | or | 6.58% |

Defects, whether due to heredity or to environment, are multitudinous. Even feeble-mindedness is no simple thing; some persons are born fools, some acquire foolishness, and some have foolishness thrust upon them. Even with apparently good heredity and good environment the children of many excellent people are more or less defective, and at present we know of no means to reduce the number of such mistakes of nature. The eugenicist can sometimes "explain" such mistakes after they have occurred, though unable to predict them before the event. Indeed eugenical explanations are apt to be more convincing and accurate than eugenical prophecy; it is well to have eugenical insurance against having defective children, but it is advisable not to have all your insurance in one company.—*From December Journal of Heredity.*

---

The hospital at Fort Sam Houston, already second in size in the United States, is to be increased in efficiency by the addition of a surgical pavilion, which will cost $20,000.

---

I do not pay
Thou dost not pay
He does not pay.

We have no money
You have no money
NOBODY HAS MONEY!

I have paid
Thou hast paid
He has paid.

We have paid
You have paid
EVERYBODY HAS MONEY!—*Clipped.*

